# A Cellular Fusion Cascade Regulated by LaeA Is Required for Sclerotial Development in *Aspergillus flavus*

**DOI:** 10.3389/fmicb.2017.01925

**Published:** 2017-10-05

**Authors:** Xixi Zhao, Joseph E. Spraker, Jin Woo Bok, Thomas Velk, Zhu-Mei He, Nancy P. Keller

**Affiliations:** ^1^School of Life Sciences, Sun Yat-sen University, Guangzhou, China; ^2^Department of Medical Microbiology and Immunology, University of Wisconsin-Madison, Madison, WI, United States; ^3^Department of Plant Pathology, University of Wisconsin-Madison, Madison, WI, United States; ^4^Department of Bacteriology, University of Wisconsin-Madison, Madison, WI, United States

**Keywords:** hyphal fusion, aflatoxin, *ham-6*, *ham-9*, *nosA*, *adv-1*

## Abstract

*Aspergillus flavus* is a saprophytic soil fungus that poses a serious threat worldwide as it contaminates many food and feed crops with the carcinogenic mycotoxin called aflatoxin. This pathogen persists as sclerotia in the soil which enables fungal survival in harsh environmental conditions. Sclerotia formation by *A. flavus* depends on successful cell communication and hyphal fusion events. Loss of LaeA, a conserved developmental regulator in fungi, abolishes sclerotia formation in this species whereas overexpression (OE) of *laeA* results in enhanced sclerotia production. Here we demonstrate that sclerotia loss and inability to form heterokaryons in *A. flavus*Δ*laeA* is mediated by homologs of the *Neurospora crassa ham* (hyphal anastomosis) genes termed *hamE-I* in *A. flavus*. LaeA positively regulates *ham* gene expression and deletion of *hamF, G, H*, or *I* phenocopies Δ*laeA* as demonstrated by heterokaryon and sclerotia loss and reduced aflatoxin synthesis and virulence of these mutants. Deletion of *hamE* showed a less severe phenotype. *hamE-I* homologs are positively regulated by the clock controlled transcription factor ADV-1 in *N. crassa*. Similarly, the ADV-1 homolog NosA regulates *hamE-I* expression in *A. flavus*, is required for sclerotial development and is itself positively regulated by LaeA. We speculate that a putative LaeA>NosA>fusion cascade underlies the previously described circadian clock regulation of sclerotia production in *A. flavus.*

## Introduction

Most plant pathogenic fungi have developed both asexual and sexual modes of reproduction with specific roles in life and disease cycles. A critical developmental stage of several plant pathogenic fungi is formation of the survival structure, the sclerotium. Sclerotia are large, multicellular structures visible by the eye that are formed by branching and fusion of interwoven hyphae (Erental et al., 2008). There are three overlapping stages in the development of sclerotia: *initiation*, when hyphae begin to fusion together to form small, discrete initials; *development*, the sclerotial size enlarges with white coloration; and *maturation*, the surface becomes pigmented and harder (Willetts and Bullock, 1992). Under appropriate environmental conditions and presence of opposite mating types, sclerotia of most fungal species will develop sexual spores (Terhem and van Kan, 2014; Horn et al., 2016) which can also serve as inocula for some sclerotial producing fungi (Jurick and Rollins, 2007). Sclerotia can also germinate directly to form infective hyphae. The longevity of sclerotia in agricultural soils is a concern in crop protection and contributes to outbreaks of disease by sclerotial fungi (Coley-Smith and Cooke, 1971).

*Aspergillus flavus* is a well known sclerotia forming fungus, infamous for production of the carcinogenic mycotoxin, aflatoxin (reviewed in Amare and Keller, 2014). Field studies of the related species, *A. parasiticus*, demonstrated sclerotia inoculum was more effective than conidial inoculum in invasion of peanut pods (Horn et al., 1994). Both conidia and sclerotia contain aflatoxin (Wicklow and Shotwell, 1983) and studies have positively correlated sclerotial development with aflatoxin production (Brown et al., 2009) although one process is not necessarily dependent on the other (Chang et al., 2016, 2017). Despite the importance of sclerotia in *A. flavus* survival and pathogenesis and its correlation with toxin synthesis, little is known about the genetic program leading to sclerotia formation.

One clear example of genetic linkage of sclerotia and aflatoxin formation is demonstrated by the transcriptional regulator LaeA, a member of the conserved transcriptional regulatory Velvet Complex (Bayram et al., 2008). Deletion and overexpression of *laeA* results in loss and over production of both aflatoxin and sclerotia, respectively (Kale et al., 2008; Amaike and Keller, 2009). LaeA (Lae1) is a conserved protein in ascomycete fungi that coordinately connects secondary metabolism with sexual development (Wu et al., 2012). Similar to *laeA* loss in *A. flavus*, deletion of the *Botrytis cinerea* ortholog, BcLae1, results in loss of sclerotial production in this species (Schumacher et al., 2015). Sclerotia are the progenitor tissues of sexual stage development in *B. cinerea* (Terhem and van Kan, 2014). However, how LaeA/Lae1 regulates sclerotial formation is unknown. Other studies have implicated hyphal fusion - also known as anastomosis – as important in sclerotial formation with microscopic evidence of anastomosis events in initial developmental stages (Kohn et al., 1990; Erental et al., 2008). Loss of BcNoxD, BcNoxB, or BcNoxA, members of the NADPH oxidase complex, impair both conidial anastomosis tube (CAT) fusion and sclerotial formation in *B. cinerea* (Segmüller et al., 2008; Siegmund et al., 2015) and hyphal fusion is required for heterokaryotic sclerotia formation in *A. oryzae* (Wada et al., 2014), a non-aflatoxigenic clade of *A. flavus* (Varga et al., 2011).

Hyphal fusion is best characterized in the non-sclerotial model fungus *Neurospora crassa* (Fu et al., 2011; Herzog et al., 2015; Fleißner and Herzog, 2016; Daskalov et al., 2017), with many of the genes required for fusion events called *ham* (hyphal anastomosis) genes (Xiang et al., 2002). In *N. crassa*, MAP kinases, the striatin-interacting protein phosphatase and kinase (STRIPAK) complex and the NADPH oxidase complex, together with fungal specific proteins are wired into an intricate signaling network to mediate hyphal fusion events (Herzog et al., 2015). The MAK-2 protein and the fungal specific protein SO/HAM-1 participate in cellular crosstalk known as “ping-pong signaling” where both proteins are recruited to the plasma membrane of the growing tips in an oscillatory manner, with one phase lasting between 6 and 12 min (Fleissner et al., 2009; Serrano et al., 2017). HAM-5 functions as a scaffold-like protein proposed to link the activation of the MAK-2 cascade to upstream factors and proteins involved in hyphal fusion (Jonkers et al., 2014). HAM-2 (Xiang et al., 2002), HAM-3 and HAM-4 (Simonin et al., 2010) are members of the STRIPAK complex that governs multiple aspects of fungal development including fusion and sexual development (Dettmann et al., 2013). HAM-6, a homolog of PRO41 in *Sordaria macrospora* (Nowrousian et al., 2007), BcNoxD in *B. cinerea* (Siegmund et al., 2015) and PaNoxD in *Podospora anserina* (Lacaze et al., 2015), is an endoplasmic reticulum membrane protein and member of the NADPH oxidase complex essential for hyphal fusion in filamentous fungi. HAM-7 acts as a membrane receptor for the MAK-1 pathway during cell to cell signaling and hyphal fusion in *N. crassa* (Maddi et al., 2012). There is limited knowledge about HAM-8 and HAM-9 although phosphorylation studies showed that HAM-8 together with HAM-6 and HAM-7 regulate the MAK-1 pathway, and HAM-9 regulates crosstalk between MAK-1 and MAK-2 pathway during vegetative growth in *N. crassa* (Fu et al., 2014).

We present in this communication our finding that LaeA regulation of sclerotial formation in *A. flavus* is mediated by through transcriptional regulation of homologs of *N. crassa* fusion genes. LaeA positively regulates expression of *hamE, F, G, H*, and *I* (homologs of *ham-5, 6, 7, 8*, and *9* in *N. crassa*, respectively) with deletions of the latter four resulting in inability of *A. flavus* to form heterokaryons or sclerotia, the same phenotype as Δ*laeA*. Moreover, we find that LaeA regulation of the *ham* cascade is likely signaled through the transcription factor NosA, a homolog of the circadian regulated ADV-1 also required for cell fusion in *N. crassa* (Fu et al., 2011). NosA is required for *hamE-I* expression, cell fusion and sclerotial formation and is itself positively regulated by LaeA. Furthermore, Δ*hamF-I* and Δ*nosA* result in significantly decreased aflatoxin synthesis and decreased virulence on corn seed.

## Materials and Methods

### Strains and Culture Conditions

Strains used in this research are listed in **Table 1** and stored as glycerol stocks at -80°C. All strains were grown on glucose minimal medium (GMM) (Shimizu and Keller, 2001) for spore production at 29°C. In some cases, 0.56 g/L uracil and 1.26 g/L uridine, 1 g/L arginine or all three supplements were added and denoted as “+UU,” “+A,” or “+AUU,” respectively. YEP (6% peptone, 2% yeast extract, pH 5.8) medium, YES (6% sucrose, 2% yeast extract, pH 5.8) medium and GMM were used for Northern blot analysis. GMM + 0.1 M sorbitol was used for sclerotial formation. Heterokaryon analysis was assessed on GMM+0.25% Triton X-100 medium (Tsukasaki et al., 2014). For genomic DNA extraction, strains were grown on liquid GMM plus 0.5% yeast extract with appropriate supplements. Sorbitol minimal medium (GMM + 1.2 M sorbitol) (Lim et al., 2012) with appropriate supplements were used for transformant selection.

**Table 1:** *Aspergillus flavus* strains used in this research and their genotypes.

Strain	Genotype	Strain source
NRRL 3357	Wild type	Payne et al., 1993
TJW71.1	Δ*laeA*::*A. fumigatus pyrG*	Kale et al., 2008
TJW79.13	Δ*laeA*::*A. fumigatus pyrG, niaD^-^, A. nidulans niaD*::*A. flavus laeA*^∗^	Kale et al., 2008
TJES19.1	Δ*ku70, pyrG-*	Spraker and Keller unpublished
TJES20.1	Δ*ku70*, Δ*argB*::*A. fumigatus pyrG, pyrG-*	Pfannenstiel et al., 2017
TXZ1.2	Δ*ku70*, Δ*hamI*::*A. fumigatus pyrG, pyrG-*	This study
TXZ21.3	Δ*ku70*, Δ*argB, pyrG-*	This study
TXZ21.3.7	Δku70::*A. flavus argB*::*A. fumigatus pyrG, pyrG--*	This study
TXZ5.2	Δ*ku70*, Δ*hamE*::*A. flavus argB, pyrG-*	This study
TXZ6.2	Δ*ku70*, Δ*hamF*::*A. flavus argB, pyrG-*	This study
TXZ7.2	Δ*ku70*, Δ*hamG*::*A. flavus argB, pyrG-*	This study
TXZ8.1	Δ*ku70*, Δ*hamH*::*A. flavus argB, pyrG-*	This study
TXZ9.16	Δ*ku70*, Δ*hamI*::*A. flavus argB, pyrG-*	This study
TXZ5.2.2	Δ*ku70*::*A. fumigatus pyrG*, Δ*hamE*::*A. flavus argB, pyrG-*	This study
TXZ6.2.1	Δ*ku70*::*A. fumigatus pyrG*, Δ*hamF*::*A. flavus argB, pyrG-*	This study
TXZ7.2.1	Δ*ku70*::*A. fumigatus pyrG*, Δ*hamG*::*A. flavus argB, pyrG-*	This study
TXZ8.1.1	Δ*ku70*::*A. fumigatus pyrG*, Δ*hamH*::*A. flavus argB, pyrG-*	This study
TXZ15.1	Δ*ku70*, Δ*laeA*::*A. flavus argB, pyrG-*	This study
TXZ16.1	Δ*ku70*::*A. flavus argB*::*A. flavus laeA, pyrG-*^#^	This study
TXZ19.1	Δ*ku70*, Δ*nosA*::*A. flavus argB*,Δ*argB*::*A. fumigatus pyrG, pyrG-*	This study
TXZ20.1	Δ*ku70*, Δ*nosA*::*A. flavus argB, pyrG-*	This study
TXZ22.5	Δ*ku70, A. fumigatus pyrG*::*A. nidulans GPDA* promoter::*nosA, pyrG-*	This study

*^∗^Confirmed by northern there are two copies of *laeA* in this mutant, so we considered it as OE::*laeA*. ^#^Another *laeA* copy from wild type was inserted into the *KU70* gene locus to make as OE::*laeA*.*

### Strain Construction

All primers used for strain construction are listed in Supplementary Table S1. All PCR generated flanks ranged in size from 1 to 1.3 kb. All DNA transformation constructs were made by double joint PCR (Lim et al., 2012) or single joint PCR and transformed individually into the appropriate parental strain. Transformation of fungal strains was carried out according to the protocol of (Szewczyk et al., 2007) and (Yang et al., 2016) with the following modifications: 10^8^ spores were inoculated into 100 ml of rich growth media (2 g glucose, 0.5 g yeast extract, 100 μL trace element, 0.56 g/L uracil and 1.26 g/L uridine, 1g/L arginine) for 11 h at 37°C and 150 rpm, the mycelia were then collected through a sterile miracloth (Calbiochem), transferred to a 250 ml flask, and resuspended in 20 mL protoplast solution composed of 20 mM NaH_2_PO_4_ pH5.8, 20 mM CaCl_2_, 200 μL β-glucuronidase (85000 U/mL, Sigma), 200 mg lysing enzymes from *Trichoderma harzianum* (Sigma), 50 mg Driselase from *Basidiomycetes* sp. (Sigma) in 1.2 M NaCl. Protoplasting was performed at 100 rpm, 30°C for 5–6 h. After transformation, the protoplasts were plated on sorbitol minimal medium (above) plus appropriate supplements. At least 10 independent isolates were screened by Southern analysis and the 5′ and 3′ fragments used in the double joint or single joint PCR reaction to amplify specific gene fragments were used as probes labeled with dCTP αP^32^. Details for each primer and method to create each mutant are described below:

#### Parental Strain TXZ21.3

TXZ21.3 was created by deleting *A. fumigatus pyrG* which had been placed in the *argB* locus in strain TJES20.1 (Δ*ku70*,Δ*argB*::*A.fumigatus pyrG, pyrG*-). Primers 1 and 2 were used to amplify the *argB* 5′ flank, primers 3 and 4 were used to amplify the *argB* 3′ flank. Single joint PCR were used to amplify the fusion construct by primers 1 and 4, and the construct was transferred into TJES20.1 to get TXZ21.3. The transformants were selected on SMM+UUA with 1.5 mg/ml 5- Fluoroorotic Acid (FOA, Thermo Fisher Scientific). One of resulting double auxotrophic mutants, Δ*ku70* Δ*argB pyrG*-, called TXZ21.3, was used as the parental control for the following deletion strains. A schematic of how TXZ21.3 was derived is shown in **Supplementary Figure S1**. The southern of the TXZ21.3 can be found in **Supplementary Figure S2**.

#### Deletion of *ham E-I, nosA*, and *laeA*

Genes were deleted by replacing the target gene with *A. flavus argB* in the parental strain TXZ21.3 (Δ*ku70*,Δ*argB, pyrG*-) to create *pyrG-* single auxotrophs or TJES19.1 (Δ*ku70, pyrG*-) or TJES20.1 (Δ*ku70*,Δ*argB*::*A. fumigatus pyrG, pyrG*-) to create prototrophs. Primers 7 and 8 were used to amplify the *argB* gene from *A. flavus* wild type NRRL3357. To delete *hamE* (AFLA_095770) in TXZ21.3, primers 17 and 18 were used to amplify the *hamE* 5′ flank, primers 19 and 20 were used to amplify the *hamE* 3′ flank, then primers 15 and 16 were used to amplify the fusion construct. To delete *hamF* (AFLA_033600), primers 23 and 24 were used to amplify the *hamF* 5′ flank, primers 25 and 26 were used to amplify the *hamF* 3′ flank, then primers 21 and 22 were used to amplify the fusion construct. To delete *hamG* (AFLA_099760), primers 29 and 30 were used to amplify the *hamG* 5′ flank, primers 31 and 32 were used to amplify the *hamG* 3′ flank, then primers 27 and 28 were used to amplify the fusion construct. To delete *hamH* (AFLA_131310), primers 35 and 36 were used to amplify the *hamH* 5′ flank, primers 37 and 38 were used to amplify the *hamH* 3′ flank, then primers 33 and 34 were used to amplify the fusion construct. To delete *hamI* (AFLA_021920), primers 41 and 42 were used to amplify the *hamI* 5′ flank, primers 43 and 44 were used to amplify the *hamI* 3′ flank, then primers 39 and 40 were used to amplify the fusion construct. To delete *laeA* (AFLA_033290), primers 63 and 64 were used to amplify the *laeA* 5′ flank, primers 65 and 66 were used to amplify the *laeA* 3′ flank, then primers 61 and 62 were used to amplify the fusion construct. To delete *nosA* (AFLA_025720), primers 73 and 74 were used to amplify the *nosA* 5′ flank, primers 75 and 76 were used to amplify the *nosA* 3′ flank, then primers 71 and 72 were used to amplify the fusion construct. All constructs were individually transformed into TXZ21.3 to get deletion mutants as a *pyrG* auxotroph and the *nosA* construct was also transformed into TJES20.1 to obtain the prototrophic deletion mutant, and southern analyses are showed in **Supplementary Figure S2A**.

#### Complement Strains

TXZ21.3 was complemented to generate a prototrophic isogenic “wild type” strain TXZ21.3.7, for comparison with other mutants. To achieve this, primers 7 and 8 were used to amplify the *argB* gene from *A. flavus* NRRL 3357 genomic DNA, primers 9 and 10 were used to amplify the *A*. *fumigatus pyrG* gene from *A. fumigatus Af*293 genomic DNA, then primers 7 and 10 were used to fuse *A. flavus argB* and *A. fumigatus pyrG* together by single joint PCR. Primers 5 and 6 were used to amplify the *KU70* 5′ flank, primers 11 and 12 were used to amplify the *KU70* 3′ flank, then primers 13 and 14 were used to amplify the fusion construct by double joint PCR. The construct was transformed into TXZ21.3 to create TXZ21.3.7, southern analyses are showed in **Supplementary Figure S2B**.

Deletion strains of *hamE-H* were complemented with *A. fumigatus pyrG* into the *KU70* gene locus to created prototrophs. To achieve this, primers 45 and 46 were used to amplify the *KU70* 5′ flank, primers 47 and 48 were used to amplify the *KU70* 3′ flank, and the *A. fumigatus pyrG* gene was amplified from *A. fumigatus Af*293 genomic DNA using primers No. 61 and 62 from (Affeldt et al., 2014). Then primers 13 and 14 were used to amplify the fusion construct, and the construct have been transformed into Δ*hamE* -TXZ5.2, Δ*hamF* -TXZ6.2, Δ*hamG* -TXZ7.2, Δ*hamH* -TXZ8.1 mutants to obtain prototrophs, southern analyses are showed in **Supplementary Figure S2B**. *hamI* and *nosA* were deleted in TJES19.1 and TJES20.1 to obtain prototrophs, respectively. For *hamI* deletion in TJES19.1, primers T1 and T2 were used to amplify the *hamI* 5′ flank, primers T3 and T4 were used to amplify the *hamI* 3′ flank, and the *pyrG* marker was the same as used above for the complemented strains. Primers 39 and 40 were used to get the construct by fusion these three fragments together through double joint PCR. For the *nosA* deletion in TJES20.1, the same construct was used for as for *nosA* deletion in TXZ21.3. Southern analyses are showed in **Supplementary Figure S2A**.

#### Overexpression Mutants

Because an earlier study showed that adding an extra copy of *laeA* to the *A. flavus* genome resulted in overexpression of *laeA* transcripts (Amaike and Keller, 2009), we replicated that method here. To create an overexpression strain of *laeA*, primers 67 and 68 were used to amplify *A. flavus laeA* (including promoter, coding region and terminator) from *A. flavus* NRRL 3357 genomic DNA, then fused with the *argB* marker (the same marker gene used for gene deletion) to get the *argB-laeA* fragment by single joint PCR with primers 7 and 68. Primers 13 and 14 were used to fuse this fragment (*argB-laeA*) with *KU70* 5′ flank (primers 45 and 46) and 3′ flank (primers 47 and 48) together as a construct, and then transformed into the *KU70* gene locus in TXZ21.3 to obtain a *pyrG-* OE::*laeA* strain (TXZ16.1). For overexpression of the *nosA* gene, primers 77 and 78 were used to amplify the *A. nidulans gpdA* promoter from plasmid pJMP9.1 (Soukup et al., 2012), which was then fused with the *A. fumigatus pyrG* gene to get the *pyrG-gpdA* promoter fragment. Primers 73 and 79 were used to amplify the 5′flank for OE::*nosA*, primers 80 and 81 were used to amplify the 3′flank for OE::*nosA*. Then these two flanks were fused with the *pyrG-gpdA* promoter fragment by using primers 71 and 82, the construct was transformed into TJES19.1 to get OE::*nosA* (TXZ22.5).

### RNA Extraction and Northern Analysis

Gene expression was examined in all strains (NRRL 3357), Δ*laeA* (TJW71.1), OE::*laeA* (TJW79.13), TXZ21.3.7 (as wild type control), Δ*nosA* (TXZ19.1) and OE::*nosA* (TXZ22.5) following the same culture condition as in (Georgianna et al., 2010): 10^6^ conidia/mL were inoculated into 50 ml liquid YEP medium (aflatoxin-repressing), mycelia were collected after shaking with 250 rpm at 29°C for 24 h, washed with ddH_2_O and then transferred to 50 ml YES medium (aflatoxin-promoting), shaking with 220 rpm at 29°C for 6 and 24 h, respectively. Each strain and treatment had three replicates. Mycelia were collected, washed and lyophilized, and total RNA was extracted using QIAzol Lysis Reagent (Qiagen). 15–20 μg of RNA was loaded for northern analysis. The primers in Supplementary Table S1 were used to amplify the probes for *hamE* (primers 49 and 50), *hamF* (primers 51 and 52), *hamG* (primers 53 and 54), *hamH* (primers 55 and 56), *hamI* (primers 57 and 58), *nosA* (primers 69 and 70), and *actin* (primers 59 and 60).

### Sclerotial Formation, Aflatoxin Production, and Spore Enumeration

For sclerotial formation, 10^6^ conidia/plate were mixed with 3 ml GMM+0.1 M sorbitol medium (0.5% top agar), and poured onto 10 ml GMM+0.1 M sorbitol medium (1.5% agar), cultures were grown for 5 days at 29°C under dark, each strain had six replicates. Three plates were sprayed with 70% ethyl alcohol (EtOH) to wash away the spores, and then the plates were scanned for analysis sclerotial formation.

Aflatoxin was extracted from the other three replicates. A 14-mm-diameter core was punched from the center of each plate and homogenized in 3 mL 0.01% Tween 20. Three mL of ethyl acetate was added to each tube and the tubes were shaken vigorously and spun at 3,000 rpm for 15 min. The organic layer was collected, dried down, and resuspended in 500 μL 20% acetonitrile with 1% Formic acid (FA). Samples were filtered through an Acrodisc syringe filter with a nylon membrane (0.45 μm; Pall Corporation), and the samples were separated on a ZORBAX Eclipse XDB-C_18_ column (Agilent, 4.6 mm by 150 mm with a 5 μm particle size) by using a binary gradient of 1% (v/v) FA as solvent A and 1% FA in Acetonitrile as solvent B using a Flexar Binary Liquid Chromatography (LC) Pump (PerkinElmer) coupled to a Flexar LC Autosampler (PerkinElmer) and a Flexar Fluorescence Light (FL) Detector (PerkinElmer) with the excitation wavelength at 365 nm and the emission wavelength at 455 nm. The binary gradient started with an isocratic step at 80% A for 1 min followed by a linear gradient to 35% A in 10 min and an additional linear gradient to 100% B in 0.5 min. After each run the column was washed for 3 min using 100% B and was equilibrated for 3 min using 80% A. The flow rate was set to 1.5 mL/min. Identification and quantification of aflatoxin B_1_ was performed using Chromera Manager (PerkinElmer) by comparison to a standard curve derived from an aflatoxin B_1_ standard (Sigma–Aldrich, United States). Aflatoxin B_1_ was detected by a fluorescent detector with an emission wavelength of 455 nm and excitation wavelength of 365 nm.

Conidia production was quantified on the 3rd and 5th days of culture. Ten mL of 0.5% (w/v) top agar of GMMUU medium containing 10^6^ spores/plate were overlaid on 20 mL GMMUU agar plates, then plates were cultured at 29°C. To count conidia, 7 mm plug from each plate was homogenized in 2 mL 0.01% Tween 20 water, diluted 10-fold and counted with a hemocytometer. For each strain, conidia counts were performed using three replicates.

### Heterokaryotic Colony Assay

Hyphal fusion of *laeA, ham*, and *nosA* mutants were processed following Tsukasaki et al. (2014) with the following modification: conidia of the *argB* auxotrophic strain (TJES20.1) and *pyrG* auxotrophic strains (TJES19.1, Δ*laeA* -TXZ15.1, OE::*laeA* -TXZ16.1, Δ*hamE* -TXZ5.2, Δ*hamF* -TXZ6.2, Δ*hamG* -TXZ7.2, Δ*hamH* -TXZ8.1, Δ*hamI* -TXZ9.16, and Δ*nosA* -TXZ20.1) were collected from GMM medium supplemented with arginine or uridine/uracil as needed. Equal numbers of conidia from the *argB* auxotrophic strain and *pyrG* auxotrophic strains were mixed, and 10^7^ conidia/mL were spotted onto the agar media containing arginine (1 g/L) and uracil (5 mM)/uridine (5 mM). After the incubation at 29°C for 5 days, the newly formed conidia were collected, and 10^5^ conidia were spread onto the GMM+0.25% Triton X-100 medium (the 0.25% Triton X-100 restricts colony diameter to help with precise colony counts). Heterokaryotic colonies were counted after incubation for 3 days at 29°C.

This protocol required presence of conidia which could contain more than one nuclei. Previous work has shown that *A. flavus* conidia contain either one or two nuclei (Runa et al., 2015). We confirmed this using the nuclear stain DAPI (**Supplementary Figure S3**).

### Pathogenicity Assays

Pathogenicity assays were conducted according to a published protocol (Christensen et al., 2012) with some modifications. Corn kernels (Blue River organic hybrid) were washed in 70% EtOH for 5 min, rinsed with sterile water, and shaken in bleach (100 rpm) for 10 min. After three rinses in sterile water to remove the bleach, the kernels were blotted dry on sterile paper towels and a sterile needle was used to puncture a small hole in the embryo of each kernel. Four kernels were placed into a sterile scintillation vial, weighed, and inoculated with 200 μL of a 10^6^ conidia/mL suspension of spores in 0.01% Tween 20. A mock control inoculated with just 0.01% Tween 20 was also included. After inoculation, the vials were vortexed 5 s, the cap then loosened (to allow air circulation) and the vials were placed in a plastic box containing moist paper towels. The box was covered with Press’n Seal (Glad) and placed in a 29°C incubator with 12 h light/dark cycles (beginning on dark) for 5 days. Five replicates per strain were included.

After the 5-day period, 3 mL 0.01% Tween 20 were added to each vial, vortexed thoroughly for 1 min and then 100 μL were removed from the vials for spore enumeration using a hemocytometer. For aflatoxin extraction, 3 mL of ethyl acetate was added to the vials which were then shaken vigorously and spun at 3,000 rpm for 15 min. The organic layer was removed, dried down, and resuspended in 300 μL 20% acetonitrile with 1% FA. Then samples will be prepared as above (section of sclerotial formation) for aflatoxin HPLC analysis.

### Statistical Analysis

The GraphPad Prism software (La Jolla, CA, United States) was used for statistical analysis. Statistically significant differences were determined by an unpaired Student’s *t*-test with a two-tailed distribution and *P* < 0.05. The error bars in all figures indicate the standard error of the mean.

## Results

### LaeA Regulates *ham* Gene Expression

LaeA, although originally identified as a global regulator of fungal secondary metabolites (Bok and Keller, 2004), has since been associated with diverse fungal developmental processes including sclerotial production in *A. flavus* where loss of *laeA* (Δ*laeA*) and overexpression of *laeA* (OE::*laeA*) result in loss and overproduction of sclerotia, respectively, (Kale et al., 2008; Amaike and Keller, 2009). Sclerotia are overwintering structures formed through extensive hyphal fusion events and in *A. flavus* their formation is associated with aflatoxin synthesis. To try to gain insight into mechanism(s) by which LaeA regulates sclerotial synthesis, we re-examined previous microarray data of wild type, Δ*laeA* and OE::*laeA* strains of *A. flavus* (Georgianna et al., 2010) and identified a set of five LaeA regulated genes that are homologs of *ham* genes involved in hyphal fusion in *N. crassa.* Supplementary Table S2 shows the results of the *A. flavus* microarray where five putative *ham* genes, *hamE (AFLA_095770), hamF (AFLA_033600), hamG (AFLA_099760), hamH (AFLA_131310), hamI (AFLA_021920)* (homologs gene of *ham-5, 6, 7, 8*, and *9* in *N. crassa*, respectively) were significantly down regulated in Δ*laeA* and upregulated in OE::*laeA.* Other *N. crassa ham* homologs were not regulated by LaeA.

**FIGURE 1:**
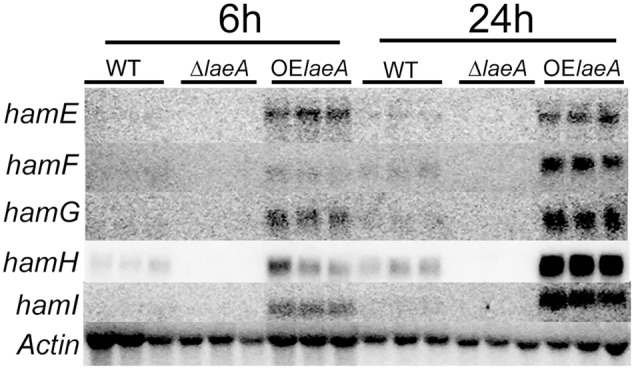
Northern result of the *ham* genes in WT, Δ*laeA* and OE::*laeA* strains. 50 ml liquid YEP medium (6% peptone, 2% yeast extract) was inoculated with 10^6^ conidia/ml of *A. flavus* NRRL3357, an *laeA* deletion strain or an *laeA* overexpression strain in 125 ml flasks, incubated with shaking at 250 rpm at 29°C. Treatments were triplicated. After 24 h, mycelia were collected and incubated in the aflatoxin-stimulating YES (6% sucrose, 2% yeast extract) medium for 6 h and 24 h (220 rpm, 29°C), respectively. *hamE* (AFLA_095770), *hamF* (AFLA_033600), *hamG* (AFLA_099760), *hamH* (AFLA_131310), *hamI* (AFLA_021920).

To confirm LaeA regulation of these five genes, RNA was extracted from the same strains – WT (NRRL3357), Δ*laeA* (TJW79.1), OE::*laeA* (TJW79.13) – and grown identically as in the microarray study (Georgianna et al., 2010) for northern blot analysis. Our results (**Figure 1**) showed that all five genes were positively regulated by LaeA with near loss in Δ*laeA* and higher expression in the OE::*laeA* strain.

### Deletion of *ham* Genes Recapitulates Δ*laeA* Phenotype of Sclerotia and Aflatoxin Loss

Studies of sclerotial formation have largely focused on morphological development, with several studies illustrating initial fusion events in sclerotia development (Kohn et al., 1990; Erental et al., 2008). We thus considered it possible that deletion of *ham* genes could decrease or eliminate sclerotial production and possibly aflatoxin synthesis due to its association with sclerotial production (Duran et al., 2006; Kale et al., 2008). *hamE, F, G*, and *H* were individually deleted in an *A. flavus* double mutant strain (TXZ21.3, Δ*KU70*,Δ*argB, pyrG-*) by replacing the *ham* encoding sequence with *argB* of *A. flavus. hamI* was deleted in an *A. flavus* single mutant strain (TJES19.1, Δ*KU70, pyrG-*) by replacing the *hamI* encoding sequence with *A. fumigatus pyrG*. Deletion mutants were confirmed by Southern blotting (**Supplementary Figure S2A**), and then one confirmed deletion mutant of each gene (excepting the *hamI* mutant as it was already a prototroph) was complemented with the *A. fumigatus pyrG* gene to create a prototroph as confirmed by Southern blotting (**Supplementary Figure S2B**).

**FIGURE 2:**
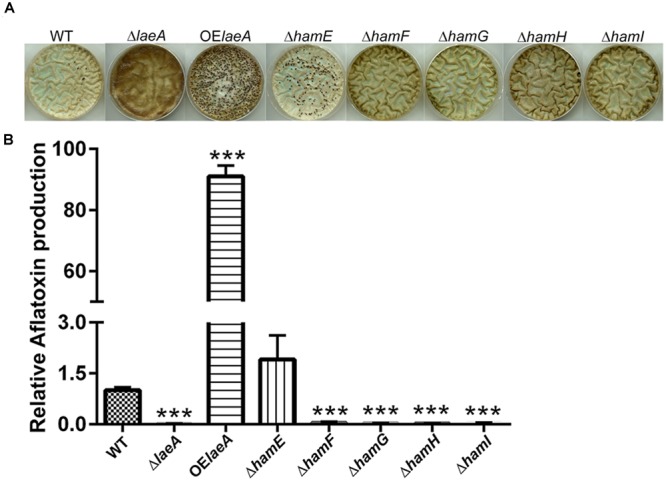
Sclerotia formation (Panel A) and aflatoxin production (Panel B) of *ham* mutants in GMM+2% sorbitol medium. **(A)** Sclerotia. **(B)** Aflatoxin. ^∗∗∗^*P* < 0.001. 10^6^ spores/plate were cultured in GMM+2% sorbitol under dark for 5 days at 29°C, spores were washed away by 70% EtOH and pictures taken to observe sclerotial formation. 1.4 cm cores were excised from each plate and extracted for analysis of aflatoxin levels via HPLC. Average of three plates for each strain.

We compared the ability of the five prototrophic Δ*ham* strains to produce sclerotia in comparison to Δ*laeA*, OE::*laeA* and wild type (TXZ21.3.7) on sclerotial inducing medium (Kale et al., 2008). As seen in **Figure 2A**, similar to Δ*laeA*, deletions of *ham F, G, H*, and *I* mutants yielded strains unable to produce sclerotia. However, the Δ*hamE* mutant still produced sclerotia in this culture condition. As reported previously, OE::*laeA* produced more sclerotia than WT (Kale et al., 2008). Aflatoxin synthesis followed that of sclerotia production on this same media with no detectable aflatoxin in Δ*laeA* and Δ*ham F-I* strains but a similar amount in Δ*hamE* as wild type and increased synthesis in OE::*laeA* (**Figure 2B**). In contrast to the effects *ham* gene loss had on sclerotial and aflatoxin production, we found no difference in asexual spore production on growth medium (**Supplementary Figure S5**).

### LaeA and HamF, G, H, and I Are Required for Heterokaryon Formation

**FIGURE 3:**
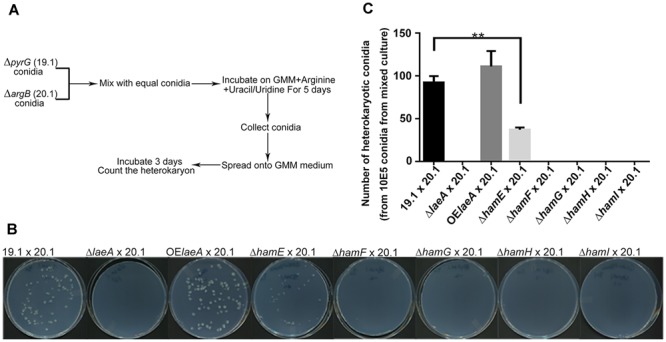
Hyphal fusion of *ham* mutants. **(A)**. Outline of the method to measure hyphal fusion in *Aspergillus oryzae* (Tsukasaki et al., 2014) which was modified for this work. **(B)** Photographs of heterokaryon formation or lack of formation in crosses. Plates of mixed cultures were spread and incubated at 29°C for 3 days. **(C)** The number of heterokaryotic colonies formed for each cross. ^∗∗^*P* < 0.01. Δ*laeA* (TXZ15.1), OE*laeA* (TXZ16.1), Δ*hamE* (TXZ5.2), Δ*hamF* (TXZ6.2), Δ*hamG* (TXZ7.2), Δ*hamH* (TXZ8.1) and Δ*hamI* (TXZ9.16) were uracil/uridine auxotrophic. 19.1 (TJES19.1), uracil/uridine auxotrophic. 20.1 (TJES20.1), arginine auxotrophic.

Heterokaryon formation also requires hyphal fusion (Glass et al., 2004) and can be the prelude to sclerotial formation between strains of the opposite mating type (Wada et al., 2014). To test for heterokaryon formation, we followed a scheme developed by Tsukasaki et al. (2014) for heterokaryon formation in the fungus *A. oryzae* with some small modifications (**Figure 3A**). Equal numbers of conidia from *pyrG* auxotrophic and *argB* auxotrophic strains were mixed together and grown on GMM medium supplemented with uracil/uridine and arginine. Conidia were collected after 5 days and spread onto GMM medium lacking supplementation where only conidia generated from heterokaryons could grow. **Figures 3B,C** illustrate successful heterokaryon formation in the *ham* and *laeA* wild type controls (TJES19.1, a *pyrG* auxotroph, crossed to TJES20.1, an *argB* auxotroph). Crosses with OE::*laeA* and Δ*hamE* mutants also led to heterokaryon formation although heterokaryon colonies were decreased in Δ*hamE* compared to both wild type and OE::*laeA*. In contrast, no heterokaryons formed in the Δ*laeA* and Δ*hamF, G, H, and I* mutants which correlated with their inability to produce sclerotia (**Figure 2**).

### Decreased Virulence of *ham* Mutants on Host Seed

**FIGURE 4:**
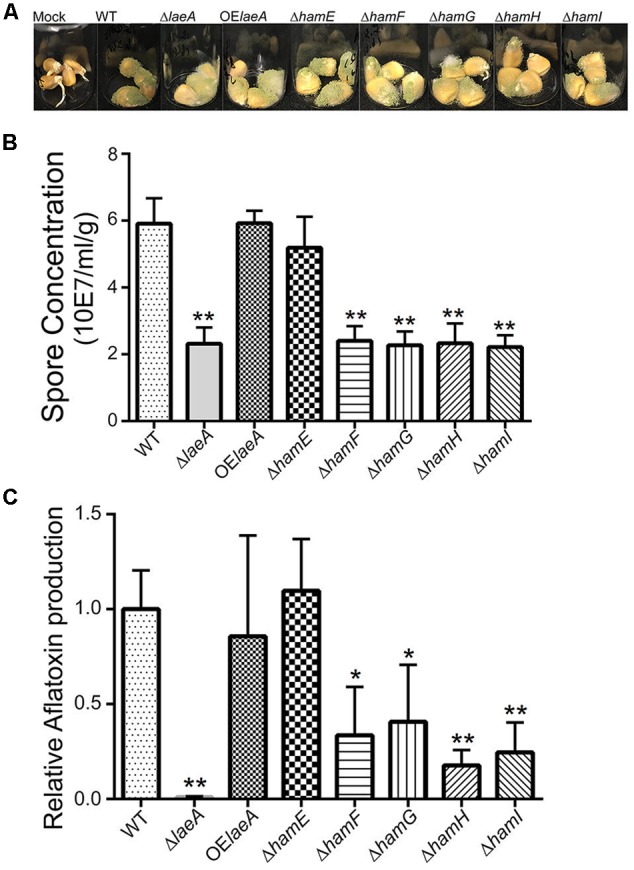
Corn infection with *ham* mutants. **(A)** 200 μl of 10^6^ spore/ml suspension of spores in 0.01% Tween 20 were inoculated on corn and compared to a mock control of 200 μl 0.01% Tween 20. The vials were kept in a moist chamber and placed in 29°C incubator with 12 h light/dark cycling and incubated for 5 days (Christensen et al., 2012). **(B)** The spores were washed off the seed with 3 ml 0.01% Tween 20 and counted. Each treatment was replicated three times. **(C)** Relative aflatoxin production of these mutants compared to WT (TXZ21.3.7). *P*-value, ^∗^*p* < 0.05, ^∗∗^*p* < 0.01.

As LaeA is required for *A. flavus* virulence on host seed, we were curious to see if loss of any *ham* gene also impacted host colonization. All five Δ*ham* mutants were compared to wild type, Δ*laeA* and OE::*laeA* in ability to colonize (as assessed by spore production) and produce aflatoxin on corn seed 5 days post inoculation. The Δ*hamE* strain presented a similar infection pattern and aflatoxin production as both wild type and OE::*laeA* (**Figure 4**). However, all the other four *ham* mutants, Δ*hamF- I*, showed reduced sporulation compared to wild type (**Figure 4B**). Aflatoxin production was reduced in Δ*hamF, G, H*, and *I* infected corn samples compared to wild type although these mutants produced slightly more aflatoxin than the Δ*laeA* mutant (**Figure 4C**).

### NosA Connects LaeA to the Ham Cascade

**FIGURE 5:**
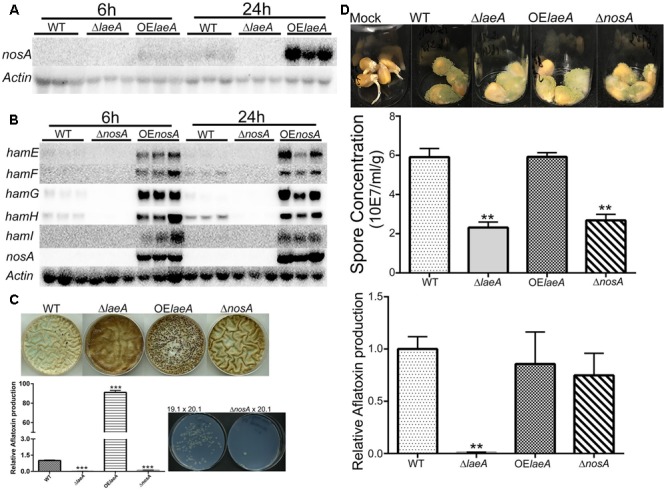
NosA regulation of *ham* genes and heterokaryon, virulence and aflatoxin production for Δ*nosA* mutants **(A)**. LaeA positively regulates *nosA* expression. The same northern blot as **Figure 1** was used for *nosA* expression. *nosA* (AFLA_025720). **(B)** NosA positively regulates *hamE-I* (using the same culture conditions as **Figure 1**). *A. flavus* WT (TXZ21.3.7), Δ*nosA* (TXZ19.1), and OE::*nosA* (TXZ22.5). **(C)** Deletion of *nosA* (TXZ19.1) results in total or near loss of sclerotia formation, aflatoxin production and heterokaryon formation. NosA deletion was assessed with the strains in **Figure 2** and the pictures of WT, Δ*laeA* and OE::*laeA* are from **Figure 2**. **(D)** Deletion of *nosA* (TXZ19.1) produces less spores but is not affected in aflatoxin biosynthesis on corn. NosA deletion was assessed with the strains in **Figure 4** and the pictures of WT, Δ*laeA* and OE::*laeA* are from **Figure 4**. Each treatment was replicated three times. *P*-value, ^∗∗^*p* < 0.01, ^∗∗∗^*p* < 0.001.

Recent studies have found that *ham-5, 6, 7, 8*, and *9*, the same subset of *ham* genes regulated by LaeA in *A. flavus*, were coordinately regulated by the transcription factor in *N. crassa* ADV-1, (Dekhang et al., 2017) and its homolog Pro1 in *S. macrospora* (Steffens et al., 2016). The orthologous transcription factor in *Aspergillus* is NosA, which is involved in sexual development in *A. nidulans* (Vienken and Fischer, 2006) and regulated by LaeA in *A. fumigatus* (Soukup et al., 2012). An examination of the *A. flavus* microarray data (Georgianna et al., 2010) showed LaeA regulated *nosA* in a similar fashion as *hamE-I* (Supplementary Table S2) which we confirmed by northern analysis (**Figure 5A**).

We next deleted and overexpressed *nosA* (**Supplementary Figure S2A**). As shown in **Figure 5B** and **Supplementary Figure S4**, *ham* gene expression was down regulated in the Δ*nosA* mutant and upregulated in OE::*nosA* mutant, respectively, in two different media. An examination of the Δ*nosA* strain showed that, similar to Δ*laeA* and Δ*hamF-I*, the mutant was unable to form heterokaryons or sclerotia and was impaired in aflatoxin synthesis on media (**Figure 5C**). The Δ*nosA* strain showed a decreased ability to infect corn (as measured by sporulation) although it showed no difference in ability to produce aflatoxin in seed (**Figure 5D**).

## Discussion

The persistence of sclerotia in adverse environmental conditions represents a clear fitness advantage for fungi. Not only can sclerotia withstand environmental extremes but they contain numerous bioactive secondary metabolites proposed to act as insect feeding deterrents (Laakso et al., 1994; Whyte et al., 1996). Despite their importance in plant pathology and potentially drug discovery (Lee et al., 2017), very little is known about the genetic pathways regulating their formation. Following leads from studies showing a requirement of LaeA (Lae1) for sclerotia production (Kale et al., 2008; Amaike and Keller, 2009; Schumacher et al., 2015), we present our findings supporting a model where LaeA regulation of a cell fusion cascade underscores a critical cellular mechanism required for sclerotial formation in fungi.

Hyphal fusion plays a key role in establishment of the mycelial colony, tissue development, heterokaryon formation and nutrition communication in filamentous fungi including *N. crassa, S. macrospora, Fusarium oxysporum*, and *B. cinerea* (Lichius and Lord, 2014; Fleißner and Serrano, 2016; Shahi et al., 2016). The majority of our genetic understanding of hyphal fusion is obtained from a series of *N. crassa* studies where genes involved in fusion events have been termed hyphal anastomosis genes, *ham* (Herzog et al., 2015; Fleißner and Herzog, 2016; Daskalov et al., 2017). Here we present data showing that five *A. flavus* homologs (HamE-I) of *N. crassa* Ham proteins (HAM-5-9) share the same regulatory cascade as in *N. crassa* with four of them (HamF-I) to exhibit clear roles in fusion events in *A. flavus*. Cellular fusion in *A. flavus* is most clearly observed in inability to form heterokaryons or sclerotia. The consequence of heterokaryon formation and sclerotial loss is also observed in decreased ability to synthesize aflatoxin in these mutants.

The HamE (HAM-5) mutant showed the least difference to wild type *A. flavus.* HAM-5 was originally isolated in a UV screen to isolate *N. crassa* mutants in hyphal fusion, as measured by loss of conidial anastomosis tube formation, or CAT formation (Aldabbous et al., 2010). More recently HAM-5 has been characterized as a putative scaffold protein for the MAK-2 MAP kinase complex, physically interacting with NRC-1, MEK-2, and MAK-2 (Jonkers et al., 2014). The protein is an important player in oscillation kinetics during CAT formation in *N. crassa* (Jonkers et al., 2014). The homolog of HAM-5 in *P. anserina*, IDC1, is a member of the PaMpk1 signaling pathway and required for the movement of a MAP kinase into the nucleus (Jamet-Vierny et al., 2007). The *A. flavus hamE* mutant was deficient in heterokaryon formation (**Figure 2, 3**) although fusion was not completely eliminated in contrast to the other four *ham* mutants. Also, this mutant was still able to produce sclerotia and aflatoxin and demonstrated equal virulence as wild type. If HamE is a member of homologous MAK-2 Map kinase pathway in *A. flavus*, these results may suggest this pathway is not heavily involved in fusion dynamics.

In contrast to HamE loss, deletion of HamF-I (HAM-6-9) showed near identical phenotypes to Δ*laeA*. HAM-6, HAM-7 and HAM-8 were all identified in a CAT fusion screen separate from the HAM-5 screen (Fu et al., 2011). Their sequence suggested they might form a cell membrane complex with experimental evidence to link such a complex to the MAK-1 cell wall integrity MAPK pathway due to a dramatic decrease of MAK-1 phosphorylation in Δ*ham-6*,Δ*ham-7*, and Δ*ham-8 N. crassa* mutants (Fu et al., 2014). However, a clear picture of such a complex still needs to be resolved. BcNoxD/Pro41/PaNoxD are the homologs of HAM-6 in *B. cinerea* (Siegmund et al., 2015), *S. macrospora* (Nowrousian et al., 2007), and *P. anserina* (Lacaze et al., 2015), respectively. These proteins are thought to be homologs of the p22phox NADPH subunit in fungi and found located at the ER (Lacaze et al., 2015; Scott, 2015). The p22phox subunit interacts with the gp91phox protein to form the NADPH oxidase complex in fungi and mutants of gp91phox display similar phenotypes as p22phox mutants. In particular, the deletion of the gp91phox equivalent in *A. nidulans*, NoxA, results in loss of sexual fruit body (cleistothecium) formation in that fungus (Lara-Ortíz et al., 2003). Cleistothecia are the developmental equivalent to sclerotia in *A. flavus*, thus fitting in with our observation of sclerotial loss in Δ*hamF* mutants of *A. flavus*. We would predict that deletion of the *noxA* homolog in *A. flavus* or deletion of *hamF* in *A. nidulans* would result in loss of sclerotia and cleistothecia in each species, respectively.

There is limited knowledge about HAM-7, HAM-8, and HAM-9. HAM-7, a GPI-anchored cell wall protein, is required for activating the MAK-1 MAP kinase cascade and regulates cell wall integrity and hyphal anastomosis (Maddi et al., 2012; Fu et al., 2014). As mentioned earlier, HAM-8 is proposed to function in this same signaling network (Fu et al., 2014). This same study suggested that HAM-9 might play slightly different roles in the network as its loss did not generate all of the same physiological patterns as HAM-6, HAM-7, and HAM-8 mutants. The authors suggested HAM-9 might mediate the communication of the two MAPK pathways during hyphal fusion (Fu et al., 2014). The phenotypes of deletions of all four *ham* equivalents (e.g., *hamF-I*) in *A. flavus* were identical in all parameters tested in this study with identity to Δ*laeA* in lack of heterokaryon fusion (**Figure 3**) and sclerotial development (**Figure 2A**) although the Δ*laeA* mutant showed a more extreme aflatoxin loss during colonization of corn (**Figure 4C**).

Relatively few studies have examined the impact of loss/impaired fusion on virulence of pathogenic fungi but, of those published, anastomosis appears to be important for virulence. Deletion of the SO homolog (Aso1) in *Alternaria brassicicola* yielded a strain lacking the ability to fuse and greatly impaired in ability to form lesions on cabbage leaves (Craven et al., 2008). The *F. verticillioides* SO mutant (FvSO) was essential for infection of corn ears, talks and seedling (Guo et al., 2015). A striatin mutant in the corn pathogen *Colletotrichum graminicola* was defective in stalk rot and leaf blight infections, showed reduced hyphal fusion and was defective in sexual development (Wang et al., 2016). The orthologous mutant in *F. verticillioides* likewise was significantly impaired in virulence (Zhang et al., 2017). The *B. cinerea noxD* (*ham-6/hamF*) mutant was less virulence when it was inoculated on French bean plants (*Phaseolus vulgaris*) (Siegmund et al., 2015). Our results support a requirement for hyphal fusion for wild type virulence of *A. flavus* on corn seed and, additionally, production of aflatoxin (**Figure 4C**).

In addition to LaeA, other parameters have been shown to regulate sclerotia production in *A. flavus* including a circadian oscillator where sclerotia production peaks at early evening (Greene et al., 2003). The *Aspergillus* clock components remain unresolved as this genus does not contain an ortholog of *frequency*, the central clock protein in *N. crassa* (Montenegro-Montero et al., 2015). Recently, the *N. crassa* clock controlled transcription factor ADV-1 (Dekhang et al., 2017), and the homolog protein Pro1 in *S. macrospora* (Steffens et al., 2016), both were shown to regulate the same *ham* genes (*ham-5*, -*6*, -*7, -8*, and *-9*) as regulated by LaeA in *A. flavus*. The work in *N. crassa* further demonstrated that ADV-1 transduces clock driven rhythmic expression of these *ham* genes and thus hyphal fusion events. The homolog of ADV-1/Pro1 in *Aspergillus*, NosA, controls fruiting body formation in *A. nidulans* (Vienken and Fischer, 2006). Intriguingly, we find *A. flavus* NosA to regulate the same five *ham* genes as ADV-1/Pro1 and LaeA and loss of Δ*nosA* to exhibit the majority of the Δ*laeA* phenotypes similarly to deletion of *hamF-I*. Furthermore, *nosA* itself is under the transcriptional control of LaeA (**Figure 5**). Thus we speculate that the circadian output of sclerotial formation in *A. flavus*, reported in Greene et al. (2003), may in part be regulated through a LaeA>NosA>fusion cascade, a topic of further investigation in our laboratory.

## Author Contributions

XZ carried out the experimentation of this work and with NK conceived experiments and wrote the manuscript. JS, JB, and TV helped create strains for this research. Z-MH helped revise this manuscript.

## Conflict of Interest Statement

The authors declare that the research was conducted in the absence of any commercial or financial relationships that could be construed as a potential conflict of interest.

## Supplementary Material

FIGURE S1 History of *Aspergillus flavus* double mutant TXZ21.3. *A. flavus* NRRL3357.5 (*pyrG-*) from He et al. (2007). *KU70* (AFLA_061470) ORF was replaced by *A. parasiticus pyrG* to get TJW149.27, then the *ku70* gene locus *pyrG* was knockout to get TJES19.1. *A. fumigatus pyrG* was used to replace the *argB* ORF in TJES19.1 to get TJES20.1, and then the *fumigatus pyrG* in the *argB* locus was deleted again to get the TXZ21.3

FIGURE S2 Southern blot analysis of the mutants in this research. **(A)** Southern blot of the deletion and overexpression strains. **(B)** Southern blot of the auxotroph mutants complement to prototroph.

FIGURE S3 DAPI stain of the Δ*nosA* and Δ*ham* mutants. Fresh spores were harvested from the strains with 0.05% Triton X-100 and centrifuged at 3000 rpm for 5 min. The supernatant was removed and conidia were washed twice with 1X Phosphate Buffered Saline (PBS). The pellet was re-suspended with 200 μl DAPI stain solution. DAPI stain solution was prepared by adding 5 μl of 10-mg/ml stocks of DAPI (40, 60-diamino-2-phenylinodole, Sigma–Aldrich, United States) plus 100- μl antifade PPD (P-phenylenediamine, Sigma–Aldrich, United States, stock solution 1 ng/lL) plus 900 μl of 70% glycerol. Conidia were incubated in the solution at room temperature in the dark for 20–30 min. Stained conidia were washed twice with 1X PBS buffer and then twice with water. The conidia were observed under a fluorescence microscope (Zeiss AxioMagerA10) using a DAPI filter set. **(A)** Δ*nosA* (TXZ20.1). **(B)** Δ*hamE* (TXZ5.2). **(C)** Δ*hamF* (TXZ6.2). **(D)** Δ*hamG* (TXZ7.2). **(E)** Δ*hamH* (TXZ8.1). **(F)** Δ*hamI* (TXZ9.16).

FIGURE S4 NosA positively regulates *hamE-I* in GMM medium. 10^7^ spores were inoculated into 50 ml GMM liquid medium for 48 h at 220 rpm at 29°C. RNA was extracted for Northern blot analysis using the probes described in section “Materials and Methods.”

FIGURE S5 Sporulation of Δ*hamE-I* and Δ*nosA* mutants in GMMUU medium. 10 ml top agar GMMUU medium [0.5% (w/v) agar] containing 10^6^ spores were overlaid on 20 ml GMMUU agar plates, then plates were inoculated at 29°C. The spores were counted from cores taken on 3 and 5 days separately.
