# Supplementary figures and images for: A Cellular Fusion Cascade Regulated by LaeA Is Required for Sclerotial Development in Aspergillus flavus
Source: Front Microbiol. 2017 Oct 5;8:1925. doi: 10.3389/fmicb.2017.01925 (PMC5633613; doi:10.3389/fmicb.2017.01925)

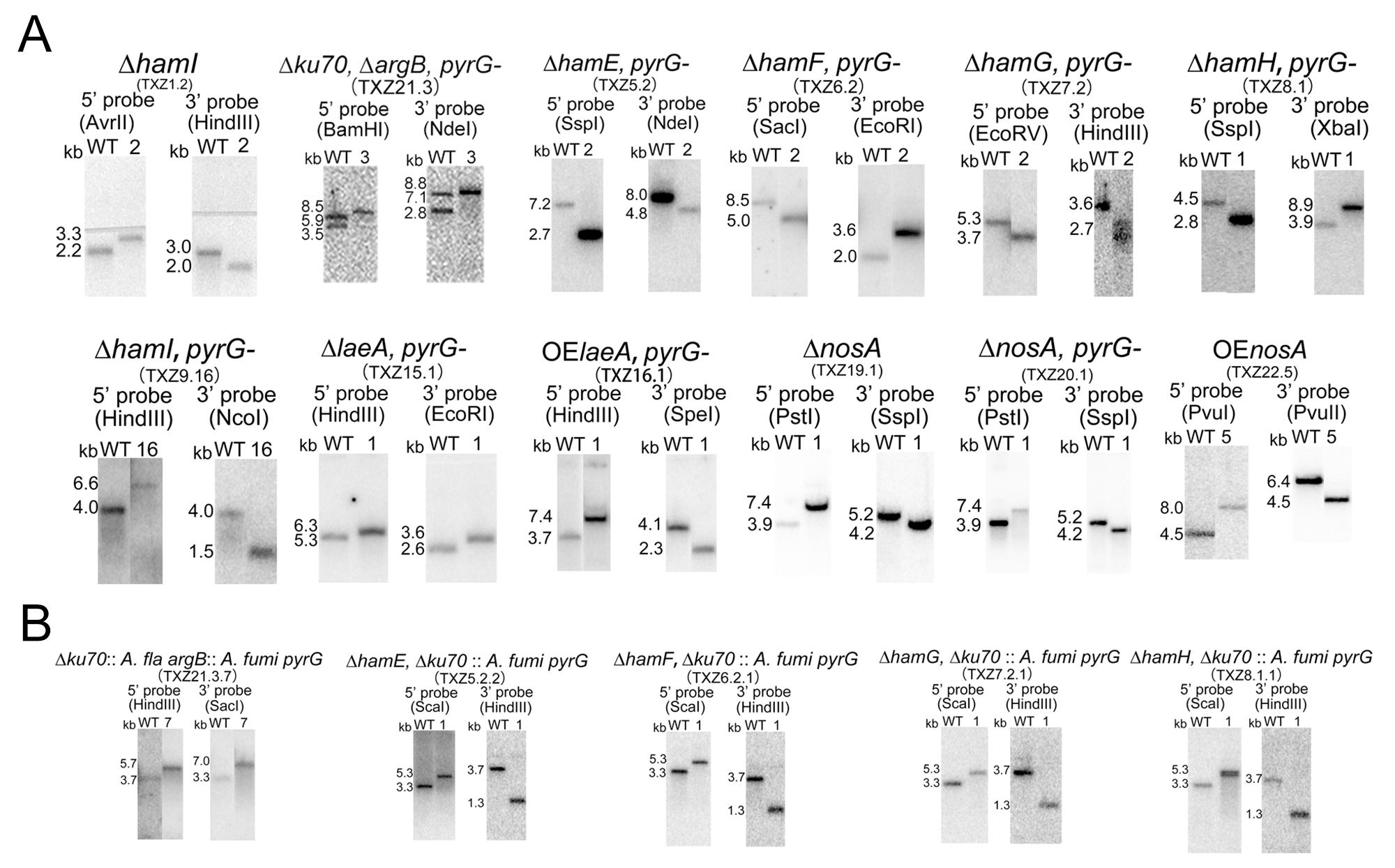

Supplement: FIGURE S2 — Southern blot analysis of the mutants in this research. (A) Southern blot of the deletion and overexpression strains. (B) Southern blot of the auxotroph mutants complement to prototroph. [file Image_2.TIF]

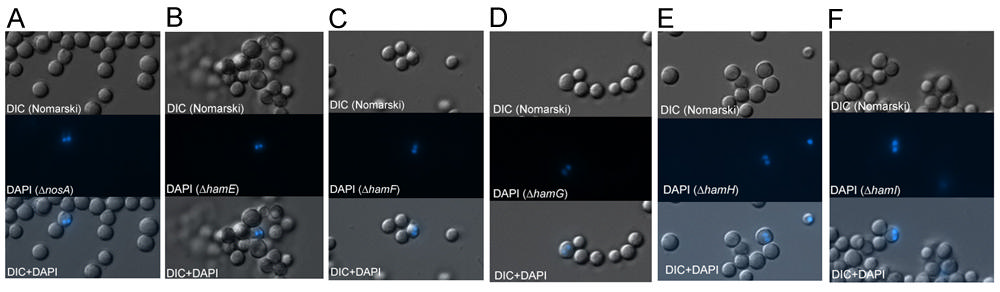

Supplement: FIGURE S3 — DAPI stain of the ΔnosA and Δham mutants. Fresh spores were harvested from the strains with 0.05% Triton X-100 and centrifuged at 3000 rpm for 5 min. The supernatant was removed and conidia were washed twice with 1X Phosphate Buffered Saline (PBS). The pellet was re-suspended with 200 μl DAPI stain solution. DAPI stain solution was prepared by adding 5 μl of 10-mg/ml stocks of DAPI (40, 60-diamino-2-phenylinodole, Sigma–Aldrich, United States) plus 100- μl antifade PPD (P-phenylenediamine, Sigma–Aldrich, United States, stock solution 1 ng/lL) plus 900 μl of 70% glycerol. Conidia were incubated in the solution at room temperature in the dark for 20–30 min. Stained conidia were washed twice with 1X PBS buffer and then twice with water. The conidia were observed under a fluorescence microscope (Zeiss AxioMagerA10) using a DAPI filter set. (A) ΔnosA (TXZ20.1). (B) ΔhamE (TXZ5.2). (C) ΔhamF (TXZ6.2). (D) ΔhamG (TXZ7.2). (E) ΔhamH (TXZ8.1). (F) ΔhamI (TXZ9.16). [file Image_3.TIF]

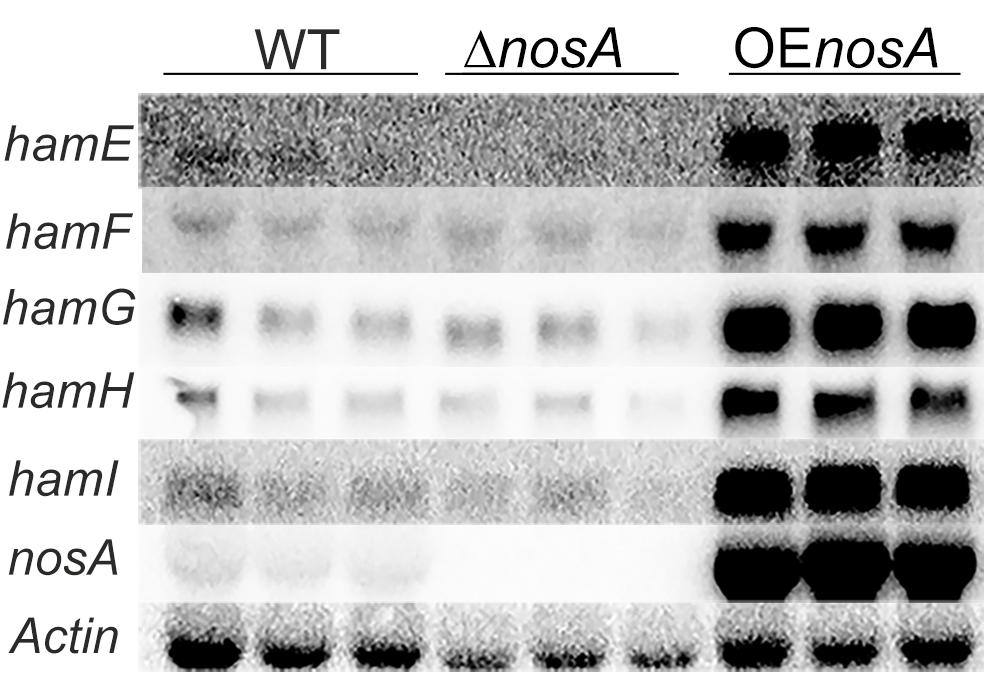

Supplement: FIGURE S4 — NosA positively regulates hamE-I in GMM medium. 107 spores were inoculated into 50 ml GMM liquid medium for 48 h at 220 rpm at 29°C. RNA was extracted for Northern blot analysis using the probes described in section “Materials and Methods.” [file Image_4.TIF]

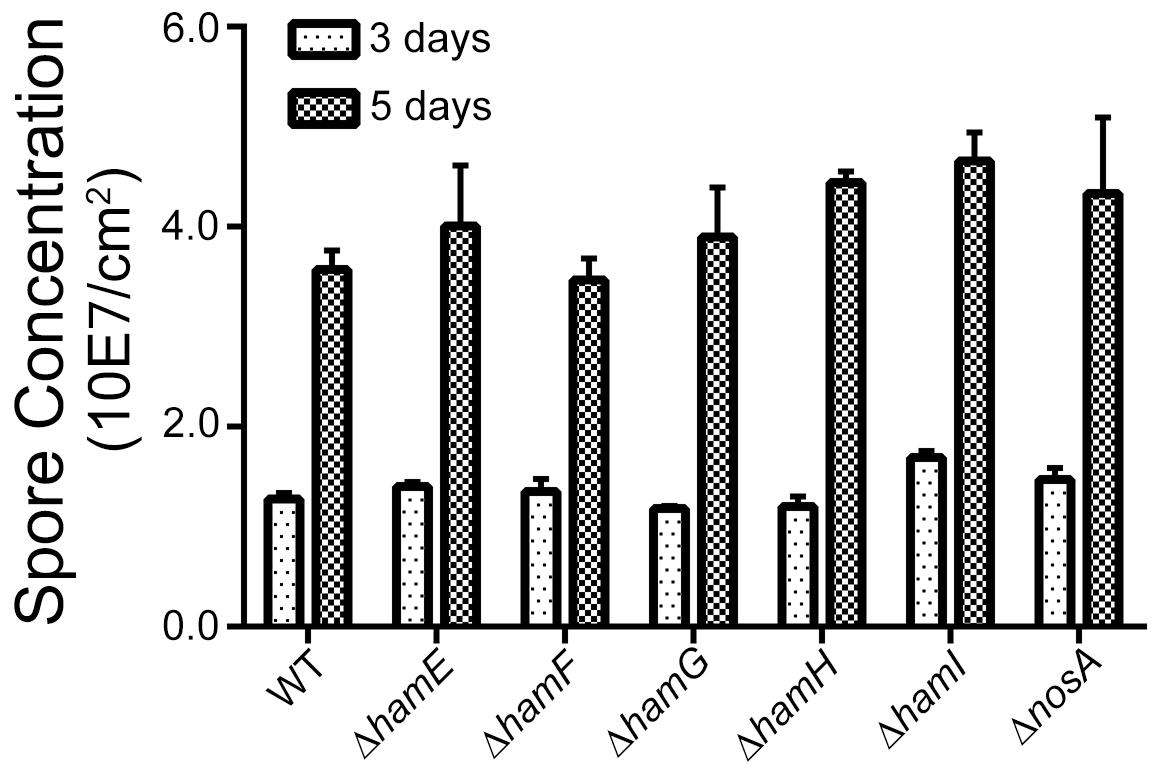

Supplement: FIGURE S5 — Sporulation of ΔhamE-I and ΔnosA mutants in GMMUU medium. 10 ml top agar GMMUU medium [0.5% (w/v) agar] containing 106 spores were overlaid on 20 ml GMMUU agar plates, then plates were inoculated at 29°C. The spores were counted from cores taken on 3 and 5 days separately. [file Image_5.TIF]
