# Supplementary material for: A Cellular Fusion Cascade Regulated by LaeA Is Required for Sclerotial Development in Aspergillus flavus
Source: Front Microbiol. 2017 Oct 5;8:1925. doi: 10.3389/fmicb.2017.01925 (PMC5633613; doi:10.3389/fmicb.2017.01925)
Supplement: Supplementary file 6 [file Table_1.docx]

**Table S1. Primers used in this research and their sequence**

| **No.** | **Name** | **Sequence (5' to 3')** |
| --- | --- | --- |
| J1 | JS_AFL_ArgBKO_F_(AfuPyrG) | CTACCCAATTGCTTCTCCGCATACTACCGGCACGATGTCGTCCAAGTCGC |
| J2 | JS_AFL_ArgBKO_R_(AfuPyrG) | GCAACTCCGCCCGTGACATGTGAATGCGGGTGAGAGGAGGCACTGATGCG |
| J3 | JS_AFLA_ArgB_5R | CCGGTAGTATGCGGAGAAGC |
| J4 | JS_AFLA_ArgB_3F | CCCGCATTCACATGTCACGG |
| 1 | JS_AFLA_ArgB_5F | ACGACCAAGATTCCGCACCG |
| 2 | JS_ArgBPyrG_5'R_recy | GCAACTCCGCCCGTGACATGTGAATGCGGGCCGGTAGTATGCGGAGAAGC |
| 3 | JS_ArgBPyrG_3'F_recy | CTACCCAATTGCTTCTCCGCATACTACCGGCCCGCATTCACATGTCACGG |
| 4 | JS_AFLA_ArgB_3R | AATCCGGGACGAAGTGTCGC |
| 5 | XZ-KU70-5'fk-F | AGAAACCCCACCATGAGGGC |
| 6 | XZ-KU70-5'flk-argB R | CCCCTTCTACCGAACTCATCACCACCGGGATAGTGGGAGGTTTGTCACCG |
| 7 | XZ-argB F | TCCCGGTGGTGATGAGTTCG |
| 8 | XZ-argB R | CCCGTGACATGTGAATGCGG |
| 9 | XZ-compl-213-pyrG F | CCGTTGGTGCCCGCATTCACATGTCACGGGTGCCTCAAACAATGCTCTTC |
| 10 | XZ-compl-213-pyrG R | GGCAGCAGAGCGACAGGCAGACACCTAGGACCAGGTATCGTCGGGAGGTA |
| 11 | XZ-KU70-3'fk F | TCCTAGGTGTCTGCCTGTCG |
| 12 | XZ-KU70-3'fk R | TCCAGCAATTGGCGCGTTGG |
| 13 | XZ-KU70-comp NF | CAATCGCTTTCCTCCATGCC |
| 14 | XZ-KU70-comp NR | ACTGCCTGTAGTCGAACAGG |
| 15 | XZ-hamE NF | ACATTTCCCGCCCAGTGCAG |
| 16 | XZ-hamE NR | GGGCGCAACGTTTTCGAGTG |
| 17 | XZ-hamE-5'flank F | ACCGTTCCTTCTCCCTTCCC |
| 18 | XZ-hamE-5'flank R+argB R | TCCCCTTCTACCGAACTCATCACCACCGGGAGCTTCCGGTTGCACCGGCA |
| 19 | XZ-hamE-3'flank F+argB F | CCGTTGGTGCCCGCATTCACATGTCACGGGGTCATTTTAATTCTATTCGC |
| 20 | XZ-hamE-3'flank R | CCGGGGGAGATTATACAGGC |
| 21 | XZ-hamF NF | TCTAGTGCAGTGGCTGGTGG |
| 22 | XZ-hamF NR | AGAAGGGTAAGGTTCGGCGG |
| 23 | XZ-hamF-5'flank F | CGAGGGTTTTGGACTGCTCG |
| 24 | XZ-hamF-5'flank R+argB R | CCCCTTCTACCGAACTCATCACCACCGGGAGCCGCGGCCGTGAGGATTATCA |
| 25 | XZ-hamF-3'flank F+argB F | CCGTTGGTGCCCGCATTCACATGTCACGGGTGACTATTTGAGTGGTTATC |
| 26 | XZ-hamF-3'flank R | TGCCAGACCAATGGAGGAGC |
| 27 | XZ-hamG NF | ATCCACTTTCACCGGCGCAG |
| 28 | XZ-hamG NR | CTCCTGGCGCAAAGTCATCC |
| 29 | XZ-hamG-5'flank F | TCTACCCGCCTGTAGAAGGG |
| 30 | XZ-hamG-5'flank R+argB R | CCCCTTCTACCGAACTCATCACCACCGGGAGGTGTCCAGTATGAGGCTCG |
| 31 | XZ-hamG-3'flank F+argB F | GCCGTTGGTGCCCGCATTCACATGTCACGGGATGCTTATGTTTCTCTTCC |
| 32 | XZ-hamG-3'flank R | AATCCAACCCCGACCCCAAC |
| 33 | XZ-hamH NF | CCTATAGCCGCTATTGCCAC |
| 34 | XZ-hamH NR | AAACTCAGCAGGGGACGCTG |
| 35 | XZ-hamH-5'flank F | GCAGACGCCTTCCGATGAAC |
| 36 | XZ-hamH-5'flank R+argB R | CCCCTTCTACCGAACTCATCACCACCGGGATGAATGAAGGCCGCGTCCAC |
| 37 | XZ-hamH-3'flank F+argB F | CCGTTGGTGCCCGCATTCACATGTCACGGGTGATTTTGACGTTTAAATAG |
| 38 | XZ-hamH-3'flank R | GATGATGGCCTTGCGTTCCC |
| 39 | XZ-hamI NF | GTTTCTCCGCTCATTAGGTG |
| 40 | XZ-hamI NR | TCCAGGAACTTAGGAGCTGG |
| 41 | XZ-hamI-5'flank F | ACAGCCTGTTGAGGTGTGCC |
| 42 | XZ-hamI-5'flank+argB R | GAGATAATTTCCGGTAGTATGCGGAGAAGCGAAGTTGCATCTGGGGTGGC |
| 43 | XZ-hamI-3'flank+argB F | GCCGTTGGTGCCCGCATTCACATGTCACGGAGATACCCATTCAACATGCC |
| 44 | XZ-hamI-3'flank R | TTTCCAGCGTGGGTACTCCC |
| 45 | XZ-KU70-5'flank-F | AGAAACCCCACCATGAGGGC |
| 46 | XZ-KU70-5'flank+AfupyrG R | CGAAGAGGGTGAAGAGCATTGTTTGAGGCATAGTGGGAGGTTTGTCACCG |
| 47 | XZ-KU70-3'flank+AfupyrG F | GTGACGACAATACCTCCCGACGATACCTGGTCCTAGGTGTCTGCCTGTCG |
| 48 | XZ-KU70-3'flank R | TCCAGCAATTGGCGCGTTGG |
| 49 | XZ-hamE-F-nor | AGGGGAATCTGACACGGACC |
| 50 | XZ-hamE-R-nor | TTGGACTGCGGTTTGCGGAC |
| 51 | XZ-hamF-F-nor | TCCACAGATCAAGTAGGCAG |
| 52 | XZ-hamF-R-nor | GAGGATATAGGATAAGGCGG |
| 53 | XZ-hamG-F-nor | GGAGGTATGCCCACCTCAAC |
| 54 | XZ-hamG-R-nor | CATAGCACCCATCACAGCCG |
| 55 | XZ-hamH-F-nor | CGAATTAAACTACGCGTCCG |
| 56 | XZ-hamH-R-nor | TTGATGCCGGACCAGAAGCC |
| 57 | XZ-hamI-F-nor | TACAAGAGGACCGTCCAGGC |
| 58 | XZ-hamI-R-nor | AAGGAGATTTCTCCGTTCCC |
| 59 | XZ-Actin-F-nor | TGAGGCACAGTCCAAGCGTG |
| 60 | XZ-Actin-R-nor | CGTGGATACCACCGCTTTCC |
| 61 | XZ-laeA-NF | GACAAGACAGCGGCTGCAAC |
| 62 | XZ-laeA-NR | TCTTGGGTCATTGGGTGGGC |
| 63 | XZ-laeA-5'flank F | CATCCCAGCGTTGCTTACGC |
| 64 | XZ-laeA-5'flank R+argB R | TCCCCTTCTACCGAACTCATCACCACCGGGATCGTCCGTTTTCTTCATAG |
| 65 | XZ-laeA-3'flank F+argB F | GCCGTTGGTGCCCGCATTCACATGTCACGGGTTCTTCTACCAACATGCGC |
| 66 | XZ-laeA-3'flank R | GAGCCCCACAAAAGCGGTAC |
| 67 | XZ-OElaeA-F | CCGTTGGTGCCCGCATTCACATGTCACGGGTAGGCTCAGCCCCATTCTAC |
| 68 | XZ-OElaeA-R | GGCAGCAGAGCGACAGGCAGACACCTAGGATGTTTGGCCTGGGTAATTTG |
| 69 | XZ-nosA-nor F | CTCATCTGCCAACACCAGCC |
| 70 | XZ-nosA-nor R | TTCCCAAGGCCGAAACGTGG |
| 71 | XZ-nosA-NF | GTGAGACGTCCGAGTACCAC |
| 72 | XZ-nosA-NR | TAGTCATGGAGAGGTGCGGG |
| 73 | XZ-nosA-5flank F | TGGGGAAAGGGAAGCTTGGC |
| 74 | XZ-nosA-5flank+argB R | CCCCTTCTACCGAACTCATCACCACCGGGATTTGGCGAGTGACTCGAGGG |
| 75 | XZ-nosA-3flank+argB F | CGTTGGTGCCCGCATTCACATGTCACGGGGTAGATACCCCCTCTCTCTCC |
| 76 | XZ-nosA-3flank R | CAACTCGGGACAACAACGGC |
| 77 | XZ-GPDA pro (+AfupyrG)-F | GTGACGACAATACCTCCCGACGATACCTGGAATTCCATCCGGATGTCGAAG |
| 78 | XZ-GPDA pro-R | GGTGATGTCTGCTCAAGCGG |
| 79 | XZ-OEnosA-5flank+AfupyrG R | CGAAGAGGGTGAAGAGCATTGTTTGAGGCATTTGGCGAGTGACTCGAGGG |
| 80 | XZ-OEnosA-3flank+GPDA pro F | ACAGCTACCCCGCTTGAGCAGACATCACCATGCCGTTGACATCGAGGAAG |
| 81 | XZ-OEnosA-3flank R | AGGGGGGAAGCATGTATGGG |
| 82 | XZ-OEnosA-NR | AGGTAGTCATCCGCAGGACC |
