# Supplementary material for: A Cellular Fusion Cascade Regulated by LaeA Is Required for Sclerotial Development in Aspergillus flavus
Source: Front Microbiol. 2017 Oct 5;8:1925. doi: 10.3389/fmicb.2017.01925 (PMC5633613; doi:10.3389/fmicb.2017.01925)
Supplement: Supplementary file 7 [file Table_2.docx]

**Table S2. *ham* and *nosA* results from the LaeA microarray data published in** (Georgianna et al., 2010)

|  |  | **DM/WT* 6h liquid** | | **DM/WT 24h liquid** | | **OE/WT* 6h liquid** | | **OE/WT 24h liquid** | |
| --- | --- | --- | --- | --- | --- | --- | --- | --- | --- |
| ***N. crassa*** | ***A. flauvs* Accession** | **Log 2** | **D-score** | **Log 2** | **D-score** | **Log 2** | **D-score** | **Log 2** | **D-score** |
| *ham-5* | AFLA_095770 (*hamE*) | -0.55 |  | -2.74 | -7.29 | 2.30 | 6.73 | 0.82 |  |
| *ham-6* | AFLA_033600 (*hamF*) | -1.43 | -3.75 | -1.64 | -5.11 | 1.20 | 3.38 | 1.02 | 7.14 |
| *ham-7* | AFLA_099760 (*hamG*) | -0.71 | -3.39 | -4.40 | -15.62 | 3.54 | 10.23 | 0.67 |  |
| *ham-8* | AFLA_131310 (*hamH*) | -1.56 | -5.08 | -5.20 | -18.88 | 3.54 | 13.93 | 0.85 |  |
| *ham-9* | AFLA_021920 (*hamI*) | -0.76 | -3.29 | -3.53 | -15.43 | 3.57 | 11.09 | 0.54 |  |
| *adv-1* | AFLA_025720 (*nosA*) | 0.18 |  | -3.48 | -15.11 | 1.38 | 3.68 | 0.74 |  |

*: DM, *A. flavus laeA* deletion mutant. OE, *A. flavus* overexpression *laeA* mutant. WT, *A. flavus* NRRL3357. Negative numbers indicate down regulation compared to WT and positive numbers indicate up regulation compared to WT at either 6 or 24 hour of growth in liquid shake medium. Details of the procedure and results are published in Georgianna et al., 2010.
